# Surgical management of penoscrotal hypospadias in a child with Opitz G/BBB syndrome: a case report

**DOI:** 10.11604/pamj.2023.44.103.38737

**Published:** 2023-02-23

**Authors:** Faisal Ahmed, Abdulfattah Altam, Qasem Alyhari, Mohamed Badheeb, Waleed Aljbri, Saleh Al-wageeh, Abdullah Al-Naggar, Saif Ghabisha, Ebrahim Al-Shami

**Affiliations:** 1 Department of Urology, School of Medicine, Ibb University of Medical Sciences, Ibb, Yemen,; 2 Department of General Surgery, School of Medicine, 21 September University, Sana'a, Yemen,; 3 Department of General Surgery, School of Medicine, Ibb University of Medical Sciences, Ibb, Yemen,; 4 Department of Internal Medicine, College of Medicine, Hadhramaut University, Mukalla, Yemen,; 5 Department of Urology, School of Medicine, 21 September University, Sana'a, Yemen,; 6 Department of Anesthesiology, Al-Thora Modern Hospital, Faculty of Medicine, Sana´a University of Medical Sciences, Sana´a, Yemen

**Keywords:** Opitz G/BBB syndrome, cleft palate, hypertelorism, penoscrotal hypospadias, case report

## Abstract

Opitz G/BBB syndrome is a rare condition characterized by three significant anomalies; hypertelorism, cleft lip and palate, and hypospadias. However, other anomalies may be associated. Herein, we report a 4-year-old child presented with penoscrotal hypospadias. On examination, hypertelorism and cleft lip and palate were noticed, suggesting a diagnosis of Opitz G/BBB syndrome. The cleft lip was corrected in the first year, and a two-staged surgical approach was implemented for penoscrotal hypospadias. In the first stage, the chordee was corrected and urethral plate was reconstructed using a tabularized incised plate urethroplasty and testicular tunica vaginalis flap. In the second stage, the remanent hypospadias was corrected, and the meatal opening reached its normal location. In conclusion, a two-staged surgical approach for the treatment of penoscrotal hypospadias associated with Opitz G/BBB syndrome may provide excellent outcomes in early-recognized cases. The urologist should pay attention to abnormal facial characteristics in patients with hypospadias.

## Introduction

The Opitz G/BBB syndrome is an uncommon genetic condition marked by several abnormalities along the body's midline [1]. Three significant anomalies have been classically recognized, hypertelorism (wide-spaced eyes), cleft lip and palate, and hypospadias However, other anomalies may be associated [2]. Opitz G/BBB syndrome is classified into two types based on genetic mutation and inheritance patterns: X-linked Opitz syndrome, which is caused by a mutation in a specific gene MID1 (midline 1) on chromosome X, and autosomal dominant Opitz G/BBB syndrome, which is caused by a deletion of 22q11.2. However, sporadic forms of this syndrome have been also reported [3]. Given the rarity of Opitz G/BBB syndrome, it can easily be unrecognized by a urologist; therefore, the report of such cases sheds light on the importance of meticulous clinical evaluation for anomalies associated with hypospadias, especially in developing countries with limited data sources [4]. Herein, we report clinical manifestation and treatment strategy in a 4-year-old Opitz G/BBB syndrome patient.

## Patient and observation

**Patient information:** a 4-year-old male child was referred to our clinic for hypospadias. The patient's history included a prior surgical repair of an incomplete cleft lip and palate at another hospital when he was one-year-old. Upon presentation, penoscrotal hypospadias with severe chordee has been noticed. There were no additional associated urinary, cardiac, respiratory, or gastrointestinal tract anomalies. No family history of hypospadias or other congenital abnormalities was reported. There was no relationship between the parents or family history of congenital anomalies. He was born at term by vaginal delivery from a 28-year-old healthy mother.

**Figure 1:**
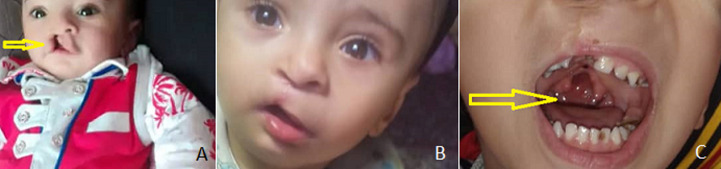
A) cleft upper lip (arrow) and widely spaced eyes; B) postoperative correction of cleft lip and epicanthus in the right eye; C) incomplete correction of cleft palate (arrow)

**Figure 2:**
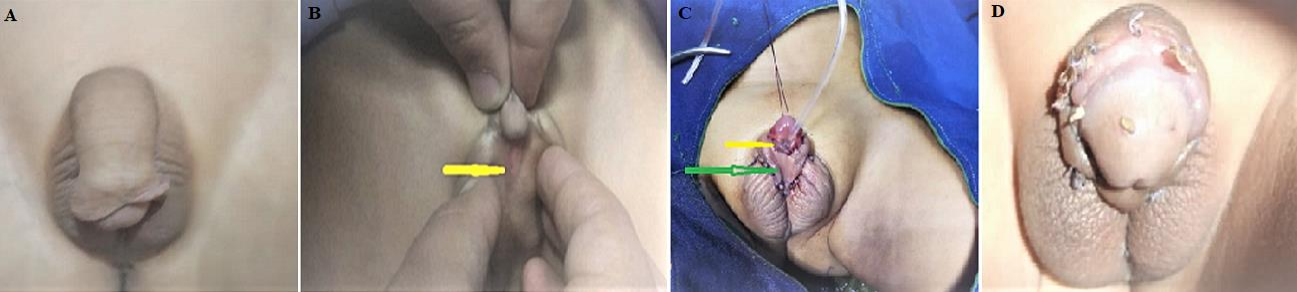
A) preoperative photo showing penoscrotal hypospadias; B) preoperative photo showing the meatal opening (arrow); C) post-operative appearance after hypospadias repair and chordee correction (yellow arrow: meatal opening, green arrow: the skin covered with the pineal midshaft buttonhole); D) the one-month postoperative appearance of the penis

**Clinical findings:** examination revealed abnormal facies consisting of mild wide-spaced, epicanthus in the right eye, and a prominent broad nasal bridge. A surgical scar was observed on the soft palate midline during the oral examination due to the incomplete cleft palate operation, in addition to another scar on the upper lip related to a previous operation (Figure 1). Examination of genitalia showed penoscrotal penile hypospadias with a severe degree of chordee and an underdeveloped scrotum. Furthermore, a hooded foreskin with rich foreskin in the dorsal aspect of the penis, absent on the ventral surface of the penis, and a penile opening in the penoscrotal area was noticed (Figure 2). There were no abnormalities in the head-neck region, trunk, or lower limbs.

**Diagnostic assessment:** all blood investigations were within normal range. Ultrasonography (US) examinations of the abdomen were normal. Echocardiography was normal. Genetic testing and karyotype analysis were not performed because they were unavailable in our city and the family poverty.

**Figure 3:**
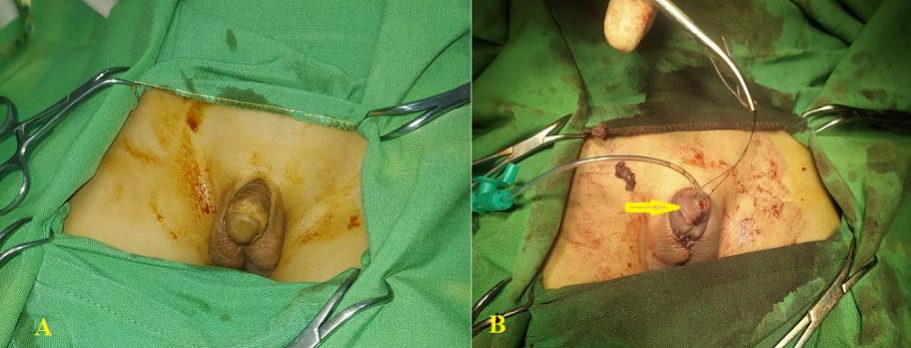
A) preoperative photo of penile appearance; B) postoperative photo showing the location of the meatal opening (arrow)

**Therapeutic interventions:** penoscrotal hypospadias was repaired in two stages. In the first stage, the chordee was repaired by total penis degloving, dysplastic dartos tissues release, and multiple transverse incisions in the penile ventral aspect. The hypospadias was corrected in the third year by tabularized incised plate urethroplasty technique and testicular tunica vaginalis flap (TVF) for neourethra coverage (Figure 2). In the second stage, the remanent hypospadias was corrected by tabularized incised plate urethroplasty technique, and the meatal opening reached its normal location in the fourth year (Figure 3).

**Follow-up and outcome:** the postoperative hypospadias surgery was eventful without complications. One-month postoperative follow-up showed an excellent penial appearance without complications or adverse events. Further surgical correction of the cleft palate was planned.

**Patient perspective:** the patient's family was pleased with the care she received throughout therapy.

**Informed consent:** written informed consent was obtained from the patient family for participation in our study.

## Discussion

Opitz *et al*. first described Opitz G/BBB syndrome (OS; Mendelian Inheritance in Man (MIM) number: 145410 and MIM number: 300000), characterized by wide-spaced eyes, hypospadias, and other anomalies in 1965 [5,6]. Opitz G/BBB syndrome has several synonyms, including Opitz oculo-genito-laryngeal syndrome, Opitz syndrome, and G syndrome [3]. However, the similar phenotypical features, mode of inheritance, and gender predominance in males endorsed a name unification; thus, Opitz G/BBB syndrome was proposed as a compound name [3]. The clinical manifestations of this syndrome are variable and range in severity from minor facial anomalies to multi-systemic congenital anomalies. Characteristically, Opitz G/BBB syndrome anomalies affect the midline in most cases, implying that the genetic mutation significantly impacts midline embryogenesis processes [7]. Opitz G/BBB syndrome has X-linked, autosomal dominant, and sporadic forms; however, no unique morphologic findings have been reported to differentiate between these hereditary forms [8]. Opitz G/BBB syndrome, also known as “hypertelorism-hypospadias syndrome,” is typically diagnosed clinically. Even within the same family, affected individuals express themselves differently. If clinical features are inconclusive, genealogical deoxyribonucleic acid (DNA) testing for a hemizygous pathogenic variant in MID1 can verify the diagnosis [4].

Major findings in this syndrome include hypertelorism (existing in almost all affected individuals). Hypospadias of varying severity, in the majority of cases, can be accompanied by congenital urinary abnormalities (85%-90%). Laryngo-tracheo-esophageal deformities include the laryngeal cleft, which can present with dysphagia and breathing dysfunction (60%-70%). Furthermore, the family history, classically, indicates an X-linked inheritance [4]. Other minor findings can be found in up to half of the patients, including learning disability and growth retardation, cleft lip, cleft palate, cardiac anomalies (e.g., ventricular and atrial septal defects, persistent left superior vena cava, persistent ductus arteriosus), anorectal malformations, brain midline defects (e.g., corpus callosum and cerebellar vermis agenesis or hypoplasia) [4,9]. In our case, the major symptoms (e.g., penoscrotal hypospadias, hypertelorism) and minor symptoms (e.g., cleft lip and palate) were presented enough to make a diagnosis. However, genetic testing and karyotype analysis were not performed because they were unavailable in our city and the family's poverty.

Hypertelorism has been identified as a common denominator in males with Opitz G/BBB syndrome. Bony interorbital distance (BIOD) was used to grade hypertelorism on plain skull radiology. Although Tessier’s classification has been used for craniofacial anomalies, including hypertelorism severity, it is implemented primarily for adult patients, as pronounced by Mulliken [10]. In affected males, hypospadias is a constant feature in 100% of the male patients, as reported by Parashar *et al.*, whereas females had no genital anomalies [2]. Cryptorchidism, however, is observed in up to one-third of cases. Hypospadias may present mildly as coronal or glandular to a more severe form as penoscrotal. The scrotum can be either normal or deft [11]. Moreover, inguinal hernias with or without genitourinary tract anomalies were reported in up to 40% of cases, Nevertheless, these anomalies were not documented in the current case [2].

Unfortunately, the characteristic facial findings of Opitz G/BBB syndrome are underrecognized, and most patients are referred to urologists or pediatric surgeons for the associated hypospadias. Therefore, a meticulous clinical evaluation should be performed for children with hypospadias, including concomitant facial anomalies such as cleft lip and palate or hypertelorism. In addition, children should be evaluated for swallowing or respiratory dysfunctions, including a history of recurrent aspirations or other laryngeal symptoms such as stridor, hoarseness, or a weak cry, as such can be fatal, and early recognition is crucial for optimal management [2]. To the best of our knowledge, this is the first documented case of Opitz G/BBB syndrome in Yemen.

The surgical approach for hypospadias repair depends on multiple factors, including the urethral meatus location, the size of the urethral plate and penis, the presence of a concomitant penile chordee, and surgical expertise. Hypospadias repair has been extensively reviewed in the literature. However, the tabularized incisional plate (TIP) has been shown to provide excellent outcomes more recently for proximal and penoscrotal hypospadias repair. The TIP procedure’s main goal is to straighten the penile chordee and move the meatus opening to the glandular area [12]. We corrected the chordee via full degloving of the penis and release of the dysplastic dartos tissues, as well as multiple circumferential incisions in the penile ventral aspect with the greatest curvature.

Tunica vaginalis flap is an excellent treatment option in penoscrotal hypospadias for neourethra coverage. Tunica vaginalis flap can be harvested through a penile incision that degloves up to the base of the penis or through an additional scrotal incision that reaches and covers the neourethra through a subcutaneous scrotal tunnel. The advantage of TVF is that it prevents future fistulas [13]. The meatal orifice location at the subcoronal area may need future cosmetic surgery, particularly if regional anatomy was not permitted to prefer expanding the neourethra up to the glandular area [13]. In our case, the second hypospadias surgery was performed to reach the metal opening into a normal position.

Our experience endorses the importance of the early recognition and repair of congenital anomalies, which can be reflected positively in patients’ quality of life, prognosis, and morbidity. Most craniofacial anomalies and hypospadias require the expertise of experienced surgeons. Therefore, these patients must be evaluated using a multidisciplinary approach, as we did in our case [1].

## Conclusion

Even though Opitz G/BBB syndrome is a rare occurrence. Identifying the simultaneous occurrence of numerous anomalies is critical for better treating patients who may require a multidisciplinary approach. Additionally, the urologist should pay attention to abnormal facial characteristics in patients with hypospadias. A two-staged surgical approach for the treatment of penoscrotal hypospadias associated with Opitz G/BBB syndrome may provide excellent outcomes in early-recognized cases.

## References

[1] Regan JP, Szymanski K, Podda S, Gargano F, Kopiecki A (2017). A surgical approach to the craniofacial defects of Opitz G/BBB syndrome. J Surg Case Rep.

[2] Parashar SY, Anderson PJ, Cox TC, McLean N, David DJ (2005). Multidisciplinary management of Opitz G BBB syndrome. Ann Plast Surg.

[3] Meroni G, Adam MP, Everman DB, Mirzaa GM, Pagon RA, Wallace SE, Bean LJH (1993). X-Linked Opitz G/BBB Syndrome. GeneReviews(®).

[4] Goraya JS, Bawa AS, Bharti S (2000). Hypospadias-hypertelorism syndrome. Indian J Pediatr.

[5] Opitz JM (1969). The BBB syndrome familial telecanthus with associated congenital anomalies. Birth Defects.

[6] Opitz JM (1969). The G syndrome of multiple congenital anomalies. Birth Defects Orig Artie Ser (V).

[7] Fernandez N, Escobar R, Zarante I (2016). Craniofacial anomalies associated with hypospadias. Description of a hospital based population in South America. Int Braz J Urol.

[8] Funke S, Kellermayer R, Czakó M, So J, Kosztolányi G, Ertl T (2006). Congenital chylothorax in Opitz G/BBB syndrome. Am J Med Genet A.

[9] Guion-Almeida ML, Richieri-Costa A (1992). CNS midline anomalies in the Opitz G/BBB syndrome: report on 12 Brazilian patients. Am J Med Genet.

[10] Tan ST, Mulliken JB (1997). Hypertelorism: nosologic analysis of 90 patients. Plast Reconstr Surg.

[11] Stevens CA, Wilroy RS (1988). The telecanthus-hypospadias syndrome. J Med Genet.

[12] Subramaniam R, Spinoit AF, Hoebeke P (2011). Hypospadias repair: an overview of the actual techniques. Semin Plast Surg.

[13] Ahmed F, Nikbakht HA, Al-Naggar K, Al-Wageeh S, Alyhari Q, Ghabisha S (2022). Role of tunica vaginalis flap and dartos flap in tubularized incisional plate for primary hypospadias repair: A retrospective monocentric study. Arch Ital Urol Androl.

